# *Drosophila* Model for the Analysis of Genesis of LIM-kinase 1-Dependent Williams-Beuren Syndrome Cognitive Phenotypes: INDELs, Transposable Elements of the Tc1/*Mariner* Superfamily and MicroRNAs

**DOI:** 10.3389/fgene.2017.00123

**Published:** 2017-09-20

**Authors:** Elena V. Savvateeva-Popova, Aleksandr V. Zhuravlev, Václav Brázda, Gennady A. Zakharov, Alena N. Kaminskaya, Anna V. Medvedeva, Ekaterina A. Nikitina, Elena V. Tokmatcheva, Julia F. Dolgaya, Dina A. Kulikova, Olga G. Zatsepina, Sergei Y. Funikov, Sergei S. Ryazansky, Michail B. Evgen‘ev

**Affiliations:** ^1^Department of Neurogenetics, Pavlov Institute of Physiology, Russian Academy of Sciences St. Petersburg, Russia; ^2^Department of Biophysical Chemistry and Molecular Oncology, Institute of Biophysics, Academy of Sciences of the Czech Republic Brno, Czechia; ^3^Department of Human and Animal Anatomy and Physiology, Herzen State Pedagogical University St. Petersburg, Russia; ^4^Department of Molecular Mechanisms of Development, Koltzov Institute of Developmental Biology, Russian Academy of Sciences Moscow, Russia; ^5^Department of Molecular Mechanisms of Biological Adaptation, Engelhardt Institute of Molecular Biology, Russian Academy of Sciences Moscow, Russia; ^6^Department of Biochemical Genetics of Animals, Institute of Molecular Genetics, Russian Academy of Sciences Moscow, Russia

**Keywords:** *Drosophila*, LIM-kinase 1, microRNA, nucleosome formation probability, transposable elements, non-canonical DNA structures

## Abstract

Genomic disorders, the syndromes with multiple manifestations, may occur sporadically due to unequal recombination in chromosomal regions with specific architecture. Therefore, each patient may carry an individual structural variant of DNA sequence (SV) with small insertions and deletions (INDELs) sometimes less than 10 bp. The transposable elements of the Tc1/*mariner* superfamily are often associated with hotspots for homologous recombination involved in human genetic disorders, such as Williams Beuren Syndromes (WBS) with LIM-kinase 1-dependent cognitive defects. The *Drosophila melanogaster* mutant *agn*^*ts*3^ has unusual architecture of the *agnostic* locus harboring *LIMK1*: it is a hotspot of chromosome breaks, ectopic contacts, underreplication, and recombination. Here, we present the analysis of *LIMK1*-containing locus sequencing data in *agn*^*ts*3^ and three *D. melanogaster* wild-type strains—*Canton-S, Berlin*, and *Oregon-R*. We found multiple strain-specific SVs, namely, single base changes and small INDEls. The specific feature of *agn*^*ts*3^ is 28 bp A/T-rich insertion in intron 1 of *LIMK1* and the insertion of mobile S-element from Tc1/*mariner* superfamily residing ~460 bp downstream *LIMK1* 3′UTR. Neither of SVs leads to amino acid substitutions in *agn*^*ts*3^ LIMK1. However, they apparently affect the nucleosome distribution, non-canonical DNA structure formation and transcriptional factors binding. Interestingly, the overall expression of miRNAs including the biomarkers for human neurological diseases, is drastically reduced in *agn*^*ts*3^ relative to the wild-type strains. Thus, *LIMK1* DNA structure *per se*, as well as the pronounced changes in total miRNAs profile, probably lead to *LIMK1* dysregulation and complex behavioral dysfunctions observed in *agn*^*ts*3^ making this mutant a simple plausible *Drosophila* model for WBS.

## Introduction

Currently, nuclear organization and 3D chromatin architecture are believed to play a main role in cognition and neuropsychiatric disorders (Medrano-Fernández and Barco, 2016).

The genomic diseases representing syndromes with multiple manifestations, occur spontaneously and sporadically as a result of contiguous deletions and duplications generated by unequal recombination in chromosomal regions with a specific architecture. Among them are the Williams Beuren syndrome, Smith-Magenis syndrome, DiGeorge syndrome (Carvalho and Lupski, 2016). These syndromes occur with a frequency of 0.7–1.0 per 1,000 live births, share neurodevelopmental phenotypes, and are detected by genome-wide segmental aneuploidy screening.

Genome-wide studies of the genomic disorders have uncovered the key role of genome architecture in the formation of structural variants (SVs). The definition of SVs overlaps with the concept of small insertions and deletions (INDELs, Mills et al., 2006). Hundreds of INDELs altering miRNA target sites of genes related to human disease pathways were identified (Bhattacharya et al., 2012). Transposable genetic elements (TEs) present an example of INDELs; upon integrating they may cause DNA insertions leading to human diseases (Ostertag and Kazazian, 2001; Mullaney et al., 2010; Carvalho and Lupski, 2016). Furthermore, miRNAs in the central nervous system are involved in epigenetic networks tuned by INDELs and TEs (Feschotte, 2008; Mattick, 2011; Morris and Mattick, 2014; Cao et al., 2016).

The DNA sequences can adopt non-B conformation, such as cruciforms, thereby affecting chromosomal structural changes in non-sequence specific manner (Hastings et al., 2009; Inagaki et al., 2009; Brázda et al., 2011; Kim and Kim, 2016). Breakpoints of gross deletions are found at the sites of non-B DNA conformation which trigger genomic rearrangements due to recombination-repair activities (Bacolla et al., 2004; Carvalho and Lupski, 2016).

Therefore, a new term “disorders of genome architecture” has been introduced to highlight a new paradigm regarding the contribution of non-B DNA structures in mutagenesis and the etiology of human genetic diseases (Wells, 2007, 2009; Kumar, 2008).

In particular, the role of chromosomal architecture is emphasized by studies of Williams- Beuren Syndrome (WBS). WBS is a neurodevelopmental disorder resulted from a heterozygous *de novo*, recurrent deletion of 26–28 genes at 7q11.23 due to nonallelic homologous recombination between large flanking low copy repeats (LCRs), including Hsmar2 transposable element from Tc1/mariner superfamily and facilitated by SVs or CNVs of the region (Pérez Jurado, 2003; Cusco et al., 2008; Gil et al., 2013). WBS is characterized by dysmorphic features, cardiovascular pathology, hypersociability, strong fixation on faces and cognitive visuospatial deficits These symptoms are often accompanied by manifestations of attention deficit hyperactivity disorder (ADHD) in WBS patients (Hoogenraad et al., 2004; Nikitina et al., 2014b and refs. therein). Since understanding the mechanisms underlying the WBS neurocognitive profile is of significant clinical importance, for a long time researchers tried to gain insights in the genomic and environmental impacts on these *de novo* events and to elucidate the role of every gene from the deleted genes (Hehir-Kwa et al., 2011). This requires development of animal models having deletions or mutations of individual genes within the WBS critical region. Such animal models are valuable to evaluate novel therapeutic approaches. Therefore, the genomic structure of the region was precisely defined in primates and mice (Valero et al., 2000). These studies have shown that although the region is inverted relative to the human map, the order of the WBS genes is conserved in the mouse genome. Moreover, each gene from WBS deletion has its Drosophila ortholog, but in this case the genes are scattered over different chromosomes (Nikitina et al., 2014a,b). LIMK1 in Drosophila belongs to the genes responsible for neurocognitive phenotype. It encodes LIM kinase 1, which affects cytoskeletal dynamics by phosphorylating and inactivating cofilin, the main regulator of actin filaments (Meng et al., 2002; Hoogenraad et al., 2004).

Previously, we have described in *D. melanogaster* the X-chromosome *agnostic* locus harboring *LIMK1* gene (Savvateeva-Popova et al., 2004; Nikitina et al., 2014b). The *agnostic* locus resides in 11AB region of the X-chromosome representing a hot spot of chromosomal breaks, ectopic contacts, underreplication in salivary gland chromosomes, and recombination (Hawley, 1980; Zhimulev et al., 1982; Xamena et al., 1985; Belyaeva et al., 1998). As a result, *agnostic* region is highly breakage-prone, and, hence, its length varies depending on a source—wild type strains *Canton-S (CS), Oregon-R* (*OrR*), or *(Ber)*, presumably due to a high rate of spontaneous unequal recombination (Savvateeva-Popova et al., 2004; Medvedeva et al., 2008; Nikitina et al., 2014a). Characteristically, these strains differ in their cognitive abilities: *Oregon-R* shows deficits in short-term memory (STM) being normal in long-term memory (LTM) retention, on the contrary, *Berlin* demonstrates LTM deficits 8 days after training. STM and LTM are drastically suppressed in *agn*^*ts*3^ relative to *CS* (Kaminskaya et al., 2012, 2015). Interestingly, *agn*^*ts*3^ shows ts-lethality at all stages of development at 29°C, but not in the adults (Savvateeva-Popova et al., 2002, 2004). Adult *agn*^*ts*3^ individuals demonstrate learning/memory defects, locomotor impairments and amyloid-like brain inclusions at normal temperature. Characteristically, heat shock treatment (HS) of *agn*^*ts*3^ adults ameliorates these mutant manifestations. While LIMK1 protein level is increased in adult *agn*^*ts*3^ brain at 22°C it drops down to that of the wild type at 29°C or 37°C (Medvedeva et al., 2008). This makes the mutation an appropriate model for gaining insights both into the genomic perturbances and environmental events provoking the *de novo* generation of WBS-linked manifestations.

Here, we report the results of *LIMK1* sequencing in *agn*^*ts*3^ and three wild-type strains that demonstrate the strain-specific SVs, namely, single base changes, small deletions, and insertions in promoter region, introns, and exons. The found INDELs may affect nucleosome formation probability (NFP) and, hence, DNA non-canonical structures formation and ectopic pairing, as well as transcription factors (TFs) and miRNAs binding. Additionally, for some reason total miRNAs expression is drastically reduced in *agn*^*ts*3^ relative to the wild-type strains.

The data accumulated herein suggest that the mechanisms of *agn*^*ts*3^ phenotype formation probably involve both *LIMK1* DNA structural rearrangements and the expression changes of certain miRNAs regulating a wide range of neurological processes.

## Materials and methods

### Fly strains

The following *D. melanogaster* strains were used:

1. wild-type strain *Canton-S* (*CS*);
2. wild-type strain *Berlin* (*Ber*) isolated from the natural population of Berlin, Germany, and widely used in European behavioral studies;
3. wild-type strain *Oregon-R* (*OrR*) isolated from the natural population of Oregon, USA, and widely used over the world as a background for marker genes and balancers;
4. *agn*^*ts*3^ mutant of the *agnostic* locus found identified in a screen for the X-chromosome-linked ethyl methanesulfonate (EMS) induced temperature-sensitive (ts) mutations affecting cAMP metabolism (Savvateeva and Kamyshev, 1981; Medvedeva and Savvateeva, 1991) on the background of *CS*. PCR mapping revealed 1.7 kb insertion ~1 kb apart from 3′-untranslated region (UTR) of the *LIMK1* gene (Medvedeva et al., 2008).

All strains were kept in 160 mL vials on the standard yeast–raisin medium at 25 ± 0.5°C and a 12:12 daily illumination cycle. Five to seven days imago males were taken in sequencing experiments.

### DNA amplification and sequencing

We have sequenced *LIMK1* gene with its 5′-untranscribed region (5′-UTR) and 3′-UTR including ~200 bp upstream exon 1 and the inter-gene spacer sequence upstream *CG1138* gene. For amplification and sequencing specific primers to *Dmel*\*LIMK1* gene (FlyBase ID: FBgn0041203) were chosen using NCBI Primer-BLAST. Fly genomic DNA (5 males per sample) was prepared using DNA extraction with DEPC according to (www.MolecularCloning.com). The gene fragments were amplified by polymerase chain reaction using Long PCR Enzyme mix (Thermo Scientific®), separated by agarose gel electrophoresis and extracted from gel using QIAquick gel extraction kit (Qiagen®). DNA sequencing was performed using Big Dye v3.1 and Big Dye v1.1 reagents (Applied Biosystems®) and 3130 Genetic Analyzer. Chromatograms were evaluated using SeqScape® Software v2.6. Genomic *LIMK1* sequence was used as a reference (GeneBank, http://www.ncbi.nlm.nih.gov/Genbank). The results of *LIMK1* sequencing in the studied Drosophila strains are submitted to GeneBank (ID: Dlimk1_CantonS—JX987486; Dlimk1_agnosticts3—JX987487; Dlimk1_Oregon-R—JX987488; Dlimk1_Berlin—JX987489).

### Heat shock treatment

A specially designed heat shock (HS) treatment protocol for *Drosophila* was used to modulate HS stress response in development and to assed its remote effects in adult 5-day-old flies (Savvateeva-Popova et al., 2008). For this, HS-treatment was applied to larvae III–pupa during formation of the central complex of the brain (critical for visual, motor, and courtship learning). The prepupae were subjected to acute heat shock in empty vials placed in a water bath for 30 min at 37°C.

### Small RNA libraries preparation

Extract RNA reagent (Evrogen, Russia) was used for total RNA extraction from adult 5 days old males. To obtain the fraction of small RNA, ~25 μg of total RNA were separated using 15% polyacrylamide gel electrophoresis in the presence of Urea (8 M) following excision of small RNA fraction corresponding to 21–29 nts. Illumina TruSeq Small RNA prep kit (Illumina, USA) was used for mall RNA libraries preparation. Sequencing was done on an Illumina HiSeq 2000 platform.

### miRNA bioinformatic analysis

Deep-sequencing results in ~10 million of small RNA reads for each library. Pre-processing procedure included: 3′-adapter cropping, filtration of reads by length filtration (>18 nt) and quality control (80% of nt should have ≥20 Phred quality). Filtered reads were mapped to the *D. melanogaster* genome (Dm3 release) by Bowtie with requiring of perfect match. The amount of mapped miRNAs reads was counted by BEDTools (v. 2.22) and mirbase annotation (r. 19) (Quinlan and Hall, 2010). Analysis of differentially expressed miRNAs was performed using edgeR (v. 3.10.2) package in R environment (v. 3.2.2) (Robinson et al., 2010).

### Computer modeling

Multiple DNA alignments were performed using SeaView software (Gouy et al., 2010). Homology-modeling of *D. melanogaster* LIMK1 protein kinase domain was performed using Swiss-Model software (Guex and Peitsch, 1997; Schwede et al., 2003; Arnold et al., 2006). IGF1R kinase catalytic domain (PDB ID: 1K3A_A) was used as a template for homology modeling. The protein 3D structures were visualized using VMD software (Humphrey et al., 1996).

The search for TF binding sites was performed using Mapper software (Marinescu et al., 2005). Additionally, the search for TF binding sites within polymorphic *LIMK1* regions was fulfilled with the help of TFSEARCH (Heinemeyer et al., 1998) and ConSite (Sandelin et al., 2004) computer resources. The search for miRNA binding sites was done using TargetScanFly resource (Ruby et al., 2007) and Segal Lab miRNA prediction tool (Kertesz et al., 2007).

The nucleosome prediction, palindrome analysis, transcription factors (TF) and miRNAs binding site search was performed for strain-specific *LIMK1* and adjacent sequences (from –223 to 8,264 bp relative to 0 bp, a *LIMK1 A/C/E* transcription start site). In the case of polymorphic nucleotides the strain-specific variants were chosen. Nucleosome prediction was made using NuPoP software (Xi et al., 2010).

DNA palindrome analysis and the search for potential DNA hairpins was performed using DNA analyzer server (Brázda et al., 2016) (Jan Kolomazník, Jiří Lýsek, Václav Brázda. DNA analyzer. Available at: http://bioinformatics.ibp.cz). The following parameter sets was used: size of palindrome: 6–500 bp, spacer: 0–1,000 bp, mismatches: 0–3.

## Results

### Comparative analysis of interstrain LIMK1 polymorphisms

**Figure 1:**
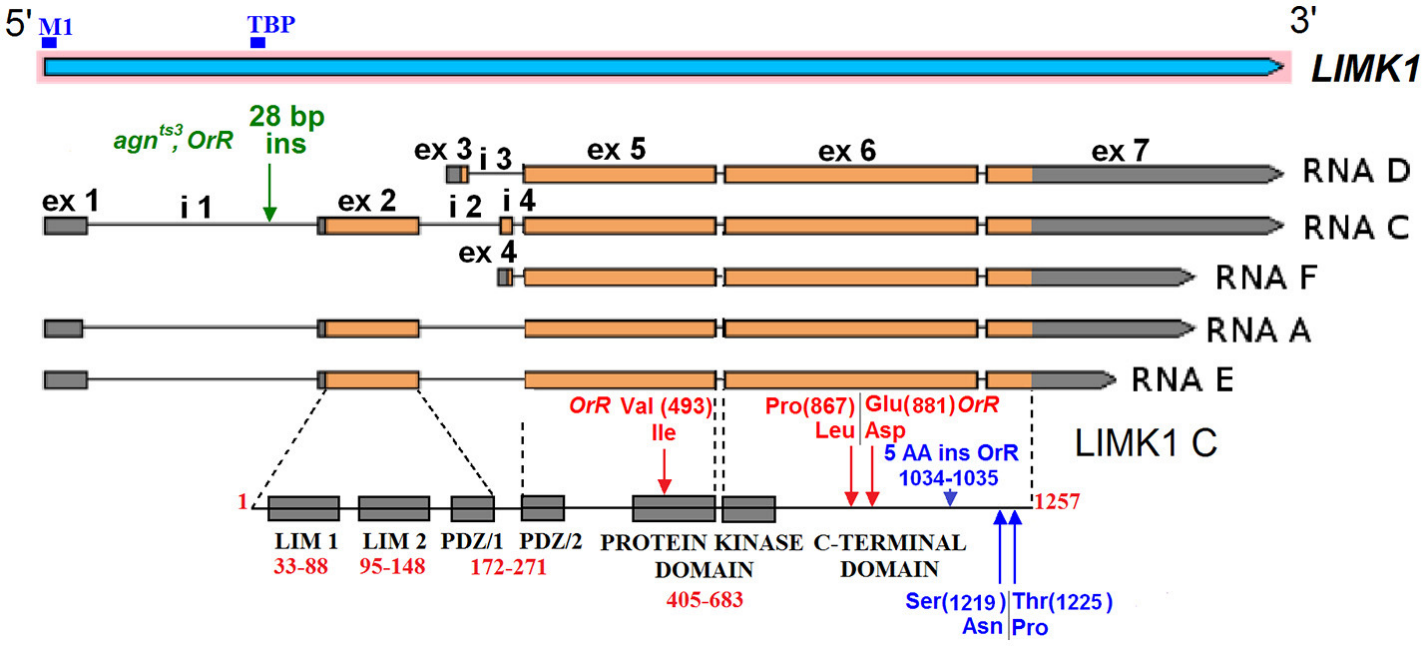
The structure of *D. melanogaster LIMK1*. The positions of exons and introns are given in accordance to FlyBase data (http://flybase.org/). The protein domain borders and the mutant amino acid residues are shown for LIMK1 C isoform. ex—exon, i—intron. Binging sites of two transcription factors: TATA-box-binding protein (TBP) and Forkhead box M1 (M1) are shown by blue bars.

*D. melanogaster LIMK1* (FBgn0041203) contains seven alternatively spliced exons, forming five different mRNA transcripts (Figure 1). *mRNA A, C* and *E* isoforms have common transcription start site (TSS) (0 bp), as well as translation start site (TlSS) (1,754 bp) within exon (1) *D/F* TSS (2510/2845 bp) and TlSS (2611/2906 bp) are within exon (2) Transcription termination sites (TTS) for different isoforms are the following: 6,767 (*E*), 7,248 bp (*A/F*), 7,808 bp (*C/D*) within exon 7. All protein isoforms have the same translation termination sites (TlTS) (6,254). *C* transcript has maximal length (5,627 bp) and *F* has minimal one (4,187 bp).

We have found strain-specific INDELs in *LIMK1* sequence in the wild-type strains (*CS, OrR, Ber*) and in *agn*^*ts*3^ (Table S1). The sequence alterations can be subdivided into several classes according to their position and functional properties: (1) mutations in promoter region; (2) mutations in introns; (3) silent mutations in exons; (4) mutations that change amino acid (AA) sequence (Tables S1, S2). *Ber* sequence is the most similar to the published genomic *LIMK1* sequence. About 20 single-nucleotide substitutions, insertions and deletions in intron 1 are common to *agn*^*ts*3^ and *OrR* and also occur in *CS* strain. The most prominent of them is A/T rich 28 bp insertion. T(785)/G polymorphism occurs only in *agn*^*ts*3^ *LIMK1*, but its functional effect is unknown.

**Figure 2:**
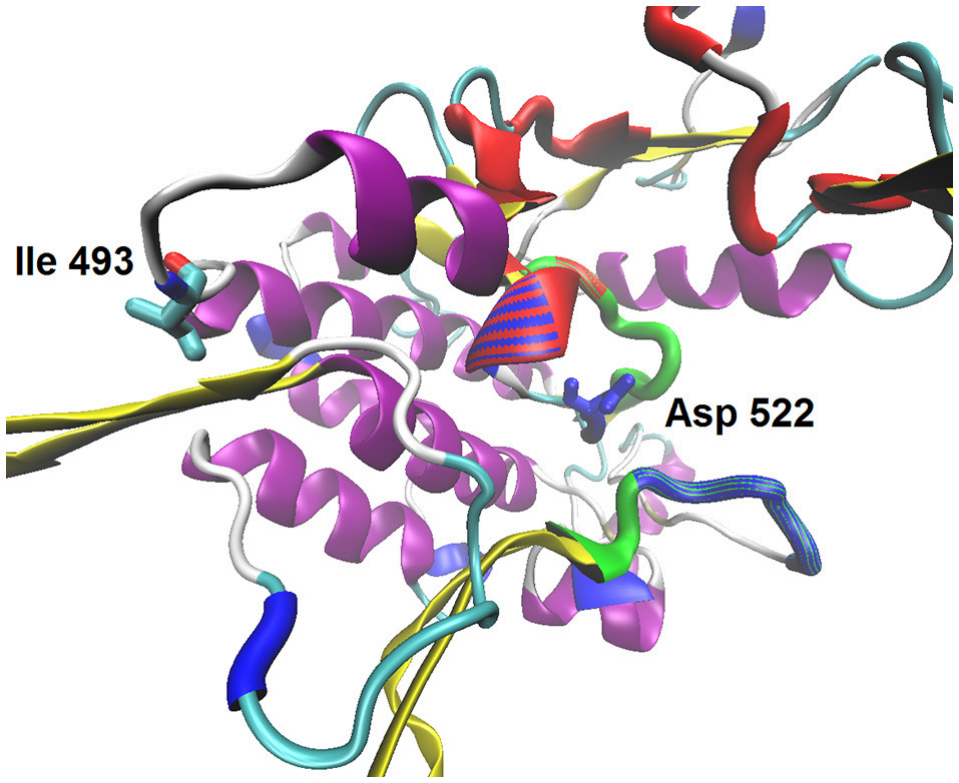
The model structure of *D. melanogaster* LIMK1 protein kinase domain. The color scheme: ATP-binding site is shown by red, the substrate binding site—by blue, the activation loop—by green; protein secondary structure: alpha-helixes are shown by magenta, beta-sheets—by yellow. Ile 493—the polymorphic residue at the external loop; Asp 522—the catalytic residue within the active side.

The gene region from exon 5 to exon 6 encodes the major part of LIMK1 protein, from the middle of PDZ domain to C-terminal domain except ~100 terminal AA residues. In this region, multiple single base polymorphisms in *CS* sequence and the corresponding base substitutions in *OrR* are observed. The majority are silent, but five of them lead to the AA polymorphisms in *CS* and substitutions in *OrR*, respectively: Val(493)Ile, Pro(867)Leu, Glu(881)Asp. (Here and thereafter: the residue numbers are given for C isoform of reference protein sequence). Val(493)Ile substitution resides in the side loop beyond the catalytic site of the LIMK1 protein kinase domain (Figure 2). *CS* LIMK1 also contains two polymorphic residues within C-terminal part: Ser(1219)Asn, and Thr(1225)Pro. *OrR* contains five additional AA in C-terminal part (1034-1035 AA: Gly Thr Ile Val Asn) due to 15 bp insertion in exon 6 and this is a strain-specific feature. Exons 5 and 6 are similar in all protein isoforms. All substitutions in A—F isoforms do not lead to ORF shift. *agn*^*ts*3^ and *Ber* exons 5 and 6 closely resemble those of genomic sequence.

The insertion of S-element (1,734 bp) from ancient Tc1/*mariner* superfamily was found in *agn*^*ts*^ (456 bp downstream *C* and *D LIMK1* TTS). *S-LIMK1* sequence is nearly identical to that of *S-AGO2{}[S-1008]* detected in the second intron of the *argonaute-2* gene (FlyBase ID: FBti0020119).

### Transcription factor binding sites search

Several possible transcription factor (TF) binding sites are found within *LIMK1* polymorphic regions (Table 1). 5′-UTR contains the binding sites for Hb and CF2-II within 100 bp upstream TSS. Substitutions A(–71)T and C(9)T are found in Hb and M1 sites of *agn*^*ts*3^ and *OrR* sequences. TATA box precedes the 28 bp A/T–rich insertion. In addition to possible TF binding sites in *LIMK1* polymorphic regions, several sequences with a high degree of similarity to TF binding sites (*E* < 1) were found in *LIMK1* and *S-LIMK1* sequences (Figure S1). Most of *LIMK1* TF sites are shared by all strains, but some are variable. *S-LIMK1* insertion creates additional 13 binding sites, such as Foxa2, Foxd3, Foxq1, and NFYA. Also, it promotes an appearance of additional br_Z1 binding sites in *agn*^*ts*3^.

**Table 1:** Some strain-specific polymorphisms in *LIMK1* sequence and their possible effects.

**Position**	**Region (region boundaries)**	**Sequences**					**Possible effects (strain)**
		**Reference**	***CS***	***agn*^*ts*3^**	***OrR***	***Ber***	
−71	71 bp upstream exon 1; Hb binding site	A	A/T	T	T	A/T	*mRNA A/C/E* transcription decrease (*agn*^*ts*3^, *OrR*)
9	Exon 1 (0–225/262); M1 binding site	C	C/T	T	T	C/T	*mRNA A/C/E* transcription decrease (*agn*^*ts*3^, *OrR*)
414–421	Intron 1 (226/263–1708)		–/8 bp del	8 bp del	8 bp del		Unknown
785	Intron 1	T	T	T/G	T	T	Unknown
1,346	Intron 1, TATA box	–	–/A	A	A	–	Transcription regulation
1,352–1,353	Intron 1	–	–/28 bp ins	28 bp ins	28 bp ins	–	Nucleosome formation probability decrease (*agn*^*ts*3^, *OrR*)
1,403–1,411	Intron 1		–/9 bp del	9 bp del	9 bp del		Unknown
2,504	Exon 2 (2510–2669); HSF binding site	T	T	C	C	T	*mRNA D* transcription decrease (*agn*^*ts*3^, *OrR*)
3,828	Exon 5 (3026–4223)	G	G/A	G	A	G	Val(493)Ile polymorphism (*CS, OrR*)
5,020	Exon 6 (4293–5883)	C	C/T	C	T	C	Pro(867)Leu polymorphism (*CS, OrR*)
5,063	Exon 6	A	A/G	A	G	A	Glu(881)Asp polymorphism (*CS, OrR*)
5,522–5,523	Exon 6	–	–	–	15 bp ins	–	5 AA insertion (1034–1035) into LIMK1 C-domain, dme-miR-7-5p site disruption (*OrR*)
6,136	Exon 7	G	G/A	G	G	G	Ser(1219)Asn polymorphism (*CS*)
6,153	Exon 7 (5944–6767)	A	A/C	A	A	A	Thr(1225)Pro polymorphism (*CS*)
8,264	456 bp downstream exon 7	–	–	*S-LIMK1*	–	–	Chromosomal architecture changes (*agn*^*ts*3^)

### LIMK1 nucleosome distribution

**Figure 3:**
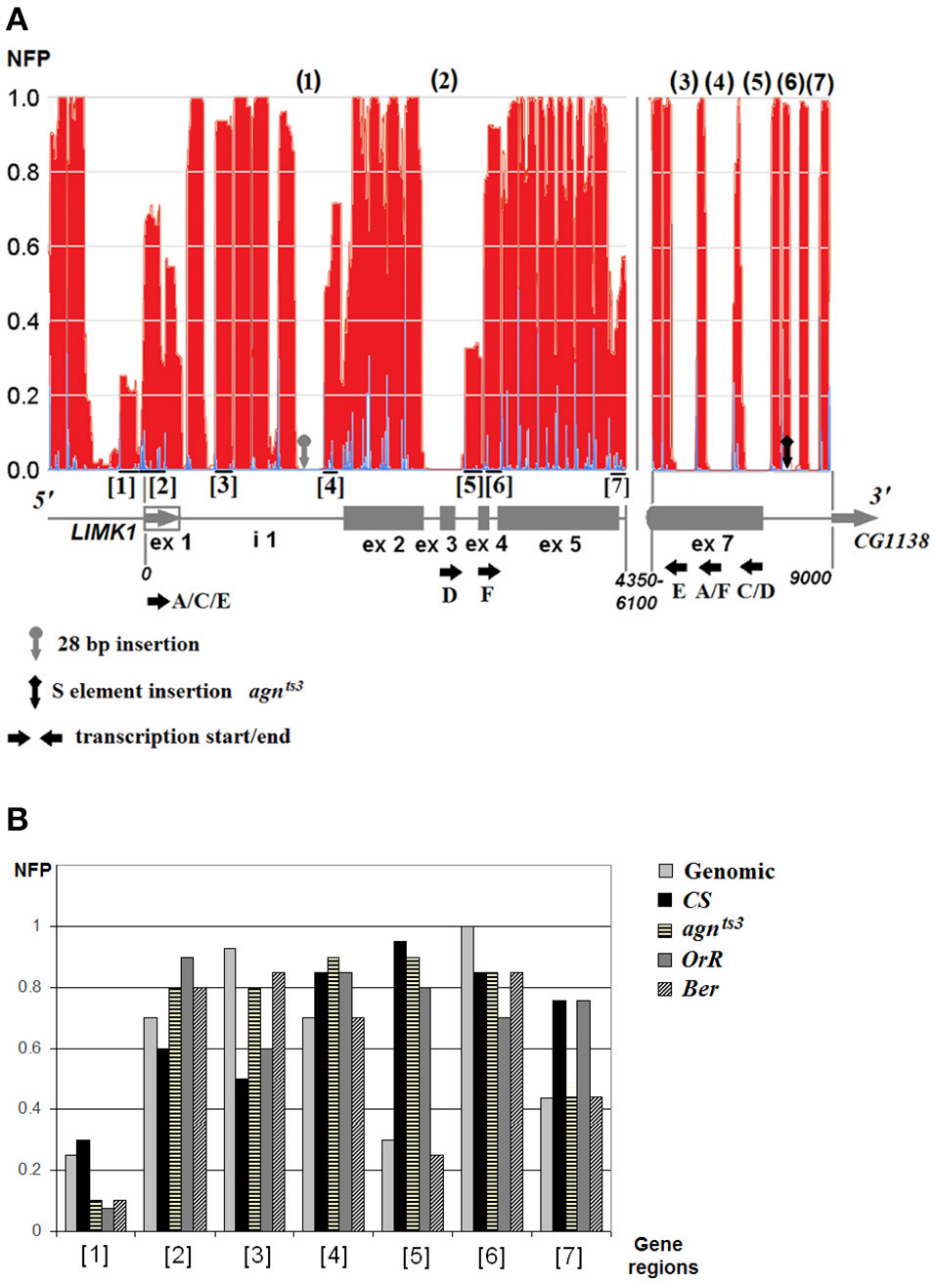
The nucleosome distribution on LIMK1 sequence. **(A)** Total nucleosome distribution on genomic LIMK1 DNA; **(B)** strain-specific NFP values. The nucleosome-free regions (NFRs) are shown by numbers in round brackets (N), the polymorphic LIMK1 regions with the nucleosomes—by numbers in square brackets [N]. The area ~4,350–6,100 in all strains is covered by nucleosomes, except small region ~4950–5075 (NFP 0.5).

Poly(A/T) tracts, among them A and A/T rich pentamers being the most frequent, are believed to disfavor nucleosome formation (Field et al., 2008). Computational analysis of NFP was performed for *LIMK1* strain-specific sequences with additional 5′–end 2,000 bp to exclude terminal effects. Positions of presumptive nucleosome start/end and the nucleosome-free regions (NFRs) appeared to be the same in all strains (Figure 3A). NFRs tend to be associated with the functional sites of *LIMK1*: D TSS (2), *mRNA E, A/F*, and *C/D* TTS (3), (4), and (5) respectively. The A/T rich 28 bp insertion within NFR(1) increases its length, thereby preventing nucleosome formation. There are a few inter-strain differences in NFP, e.g., for 5′-UTR (2) containing Forkhead box M1 TF binding site it is higher in *agn*^*ts*3^, *OrR*, and *Ber* than in *CS* (Figure 3B).

The regions with low NFP value might be of probable functional significance: *mRNA A/C/E* promoter region, *mRNA A/C/E* TSS, and *mRNA D/F* TSS (regions 1, 2, and 5, respectively). The nucleosome-free regions at 3′-end may facilitate the mRNAs transcription termination.

As to S-element, its ends are free from nucleosomes (5′: ~400 bp, 3′: ~300 bp). The insertion site of *S-LIMK1* is within a nucleosome close to its end, followed by ~120 bp NFR(6) (Figure 3). The insertion enlarges this region by ~360 bp leaving some ~400 bp nucleosome-free DNA at 3′ end of the transposon. Thus, *S-LIMK1* creates downstream *LIMK1* a large nucleosome-free region which may participate in non-homologous interactions with other NFRs, for instance A/T rich 28 bp insertion. This is the most striking feature of *agn*^*ts*3^ *LIMK1* sequence.

Northern hybridization with RNAs isolated from the wild-type strain probe revealed three transcripts: a large (3.7-kb) transcript was detectable at all stages, while two other transcripts were smaller, became detectable in third-instar larvae, and increased in size in adult females. The *agn*^*ts*3^ mutants (male third-instar larvae) showed another expression pattern (Savvateeva-Popova et al., 2004). The functional significance of this is still unclear.

### DNA palindromes and non-canonical structures

A search for DNA palindromes and potential DNA hairpins was performed within intron 1 sequence and sequence including nucleosome free regions (NFR) NFR(5)—NFR(7), with *S-LIMK1* insertion in *agn*^*ts*3^ (Table 2). The total number of palindromes for intron I mainly of 6–7 bp size is higher in *agn*^*ts*3^ and *OrR* than in annotated genomic and *Ber* sequence. In NFR(5)—NFR(7) region, *S-LIMK1* insertion leads to excess in the total number of palindromes over the sum for *S-LIMK1* and genomic sequence alone. Thus, DNA sequence downstream *agn*^*ts*3^ *LIMK1* possibly forms the non-canonical structures that may influence *LIMK1* expression.

**Table 2:** The number of palindromes in *LIMK1* intron 1 and NFR(5)–NFR(7) regions.

	**Intron 1**				**NFR(5)—NFR(7)**			
**Size**	**Genomic (1,446)**	***Ber* (1,447)**	***OrR*(1,457)**	***agn*^*ts*3^(1,456)**	**Genomic [no *S-LIMK1*] (842)**	***S-LIMK1* (1,734)**	***agn*^*ts*3^[*S-LIMK1*] (2,576)**	**Δ**
6	234	237	243	248	129	271	387	–13
7	244	237	262	260	178	335	497	–16
8	323	325	316	314	192	370	516	–46
9	310	314	298	312	167	412	587	8
10	325	330	324	326	155	384	596	57
11	221	216	216	214	102	285	444	57
12	120	122	135	129	54	206	314	54
13	66	65	67	66	15	81	134	38
14	23	22	27	27	13	44	80	23
15	14	16	18	16	6	18	30	6
16	3	3	3	3	1	7	13	5
17	2	2	2	2	2	6	9	1
18						5	5	0
Sum	1,885	1,889	1,911	1,917	1,014	2,424	3,612	174

*In brackets: DNA region length (bp). Sum – the summary number of palindromes (N)*.

*S-LIMK1**:** the sequence of S-LIMK1 without flanking DNA regions. Δ: the difference between agn^ts3^ N and the sum of genomic and S-LIMK1 Ns*.

**Figure 4:**
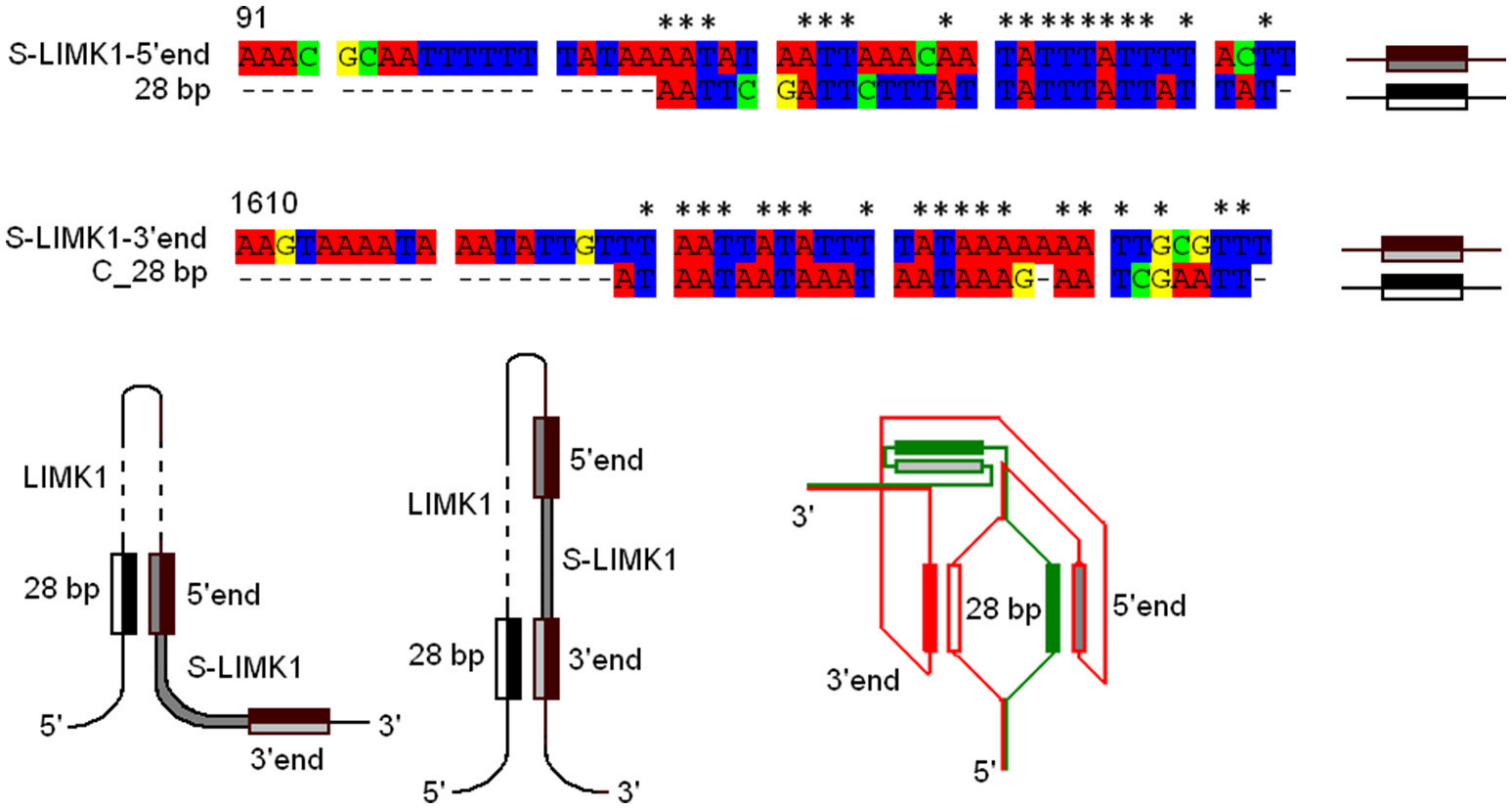
28 bp—S-LIMK1 complex: possible DNA secondary structures. The sequence of 28 bp insertion was aligned with the sequences of S-LIMK1 5′- and 3′- inverted long terminal repeats (5′ end, 3′ end). C_28 bp—the sequence of “–”DNA strand complementary to 28 bp. Nucleotide similarities are indicated by asterisks (17 in S-LIMK1 5′ and 19 in 3′). Three possible secondary structures of 28 bp—S-LIMK1 complexes are shown: 28 bp—5′ end; 28 bp—3′ end; 28 bp—5′/3′ ends. For the third structure, two DNA single strands are shown by red and green colors.

Since 28 bp insertion and *S-LIMK1* are present together only in *agn*^*ts*3^, this may explain the changes in *agn*^*ts*3^ *LIMK1* expression. The aforementioned local strand separation in superhelical DNA might be utilized due to interaction between these two insertions. Along these lines we found a partial similarity between 28 bp fragment and *S-LIMK1* (Tc1/*mariner*) terminal repeats (17 nucleotides in S-LIMK1 5′ and 19 nucleotides in S-LIMK1 3′), which may cause pairing within *LIMK1*, changing its architecture (Figure 4).

### miRNAs strain-specific expression profiles

**Figure 5:**
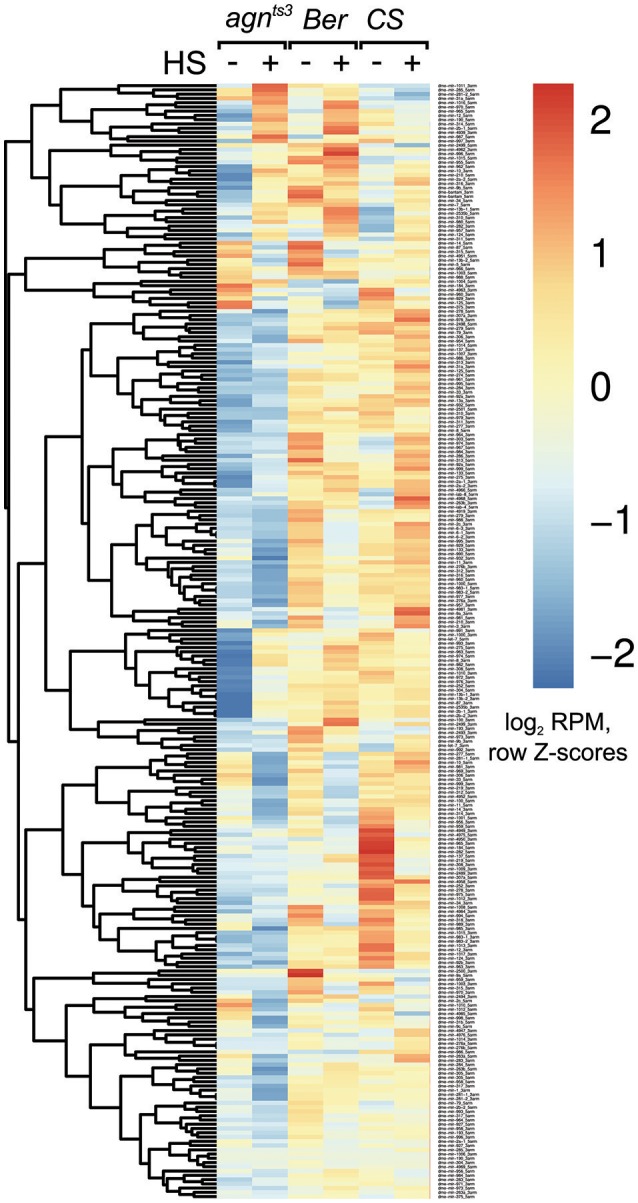
The expression of miRNAs in *agn*^*ts*3^ compared to *Berlin* (*Ber*) and *Canton- S (CS)* without (–) and after (+) heat shock (HS). The heat map represents the RPM-normalized and log2-transformed counts of miRNAs reads with *z*-scale normalization of the rows. Thirty percent of low-expressed miRNAs were removed from further analysis.

The multiple phenotypic manifestations of *agn*^*ts*3^ mutation permit to assume that it may affect some basic cellular regulatory mechanisms, in particular, the system of microRNA synthesis and processing. At present, 256 miRNA precursors and 466 mature miRNAs are known in *D. melanogaster* (miRBase 21 data) (Kozomara and Griffiths-Jones, 2014). The miRNAs expression profiles without and following HS given in development at the stage larvae III-prepupa to *agn*^*ts*^, *Ber* and *CS* and assessed in 5-day old adults are presented in Figure 5. To reveal the miRNAs with significant changes in expression after HS exposure, similarly to previously described procedure (Funikov et al., 2016) only expression changes no less than log_2_FC ≥ 1.5 were considered. Furthermore, we discarded miRNAs with <50 counts as lowly expressed.

**Figure 6:**
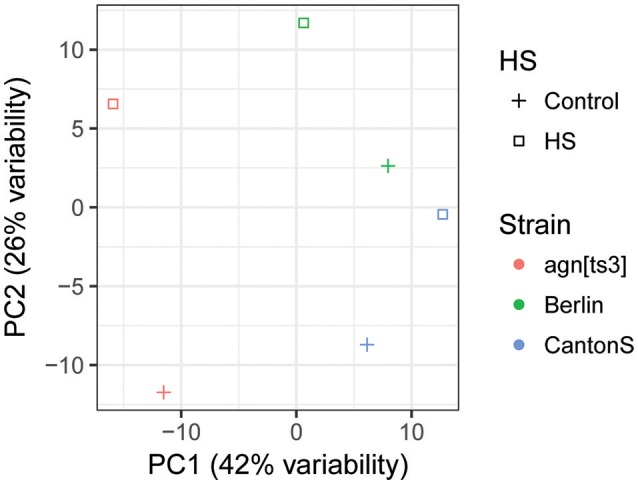
Principal component analysis (PCA) of miRNA expression for mutant *agn*^*ts*3^ and two wild-type strains—*Berlin* and *Canton-S*.

Correlation analysis and principal component analysis (PCA), were performed. It revealed a high degree of miRNA expression similarity between biological replicates (Figure 6). One can see, that *agn*^*ts*3^ demonstrates significant difference from the wild-type strains both without and after HS treatment (position on PC1 axis). At the same time the direction of miRNA expression changes after HS in *agn*^*ts*3^ is similar to *Berlin* (increasing along PC2 axis, decreasing along PC1 axis), and somewhat less similar to *Canton-S* (increasing along PC2 axis).

In *agn*^*ts*3^, 158 mature miRNAs demonstrate drastically reduced expression level relative to the other strains and as demonstrated by PCA, *agn*^*ts*3^ has a very unique miRNA expression signature. Many of them are involved in regulation of the nervous system development, behavior, and cell survival, possibly explaining the *agn*^*ts*3^—specific physiological and behavioral traits. Dramatic decrease of numerous miRNAs observed in *agn*^*ts*3^ may be due to a mutation in a component of RNA interference machinery induced by EMS treatment used in the experiments where *agn*^*ts*3^ was isolated. To check this possibility we monitored the expression of major genes involved in RNA interference in the mutant and other strains used in the study (Figure S2).

The observed diminished content of Dicer in *agn*^*ts*3^ in comparison to *Ber* might be responsible for the manifestation of such a distinctive feature of *agn*^*ts*3^, as the total decrease of miRNAs level compared to the wild-type strains. However, apparently it is not the case because the expression of Dicer 1 in *agn*^*ts*3^and *OrR* does not differ (data not shown).

### Bioinformatics analysis of *LIMK1*-miRNAs binding and miRNAs formation

Seven *D. melanogaster* miRNAs according to TargetScanFly data may interact with *LIMK1* 3'-UTR. Six of them (92a_3arm, 92b_3arm, 310_3arm—313_3arm) belong to *7mer-1A* family having the common binding site (6,685—6,691 bp).

Insertion of transposons may cause elongation of 3'-UTRs, thereby creating new miRNAs binding sites. Analysis of *D. melanogaster* miRNAs binding to *agn*^*ts*3^ *S-LIMK1* performed according to (Segal lab data; −10 kcal/mol threshold level of binding energy) demonstrates that it contains eight additional binding sites for miRNAs: 124_3arm, 312_3arm, 92_3arm, 34_5arm, 966_5arm, 87_3arm, 92b_3arm, and 1002_5arm (Figure S3).

## Discussion

During the past 35 years the convergence of DNA structural biology, genetic, and genomic studies, bioinformatics, and medicine has led to a notion that in both prokaryotes and eukaryotes segments of DNA are conformationally polymorphic. They can exist in alternative non-B DNA forms, such as, cruciforms, slipped structures, triplexes, left-handed Z-DNA, and tetraplexes. These non-B DNA conformations at specific loci of chromosomes serve as a trigger of ~20 human neurological diseases and about 50 genomic disorders. Among psychiatric diseases are schizophrenia, drug and alcohol abuse, attention-deficit hyperactivity disorder, and anorexia-bulimia. These findings have brought a new paradigm in the etiology of human genetic diseases (Wells, 2007).

It states, that although chromosomal DNA exists predominantly in right-handed B form with Watson–Crick base pairing for most of the time, different environmental events, such as temperature fluctuations and drastic changes in concentration of certain cations, radiation, etc., can cause transition of individual DNA segments from the linear B-DNA form to at least 10 non-B DNA conformations. The transition based on flexibility and plasticity of DNA may be either temporal, or long lasting. They may either cause mutations, or provide “behavioral adaptation to new experiences in a rapidly changing environment” (Medrano-Fernández and Barco, 2016). This requires short and long-range interactions between DNA sequences that are located thousands of bases apart or even in different chromosomes for coordinated regulation and implies chromatin loopings that activate regulatory sequences within discrete genomic foci (Zhang et al., 2013). Recurrent genomic copy number variants (CNVs) is a by-product of such interactions. Among the *de novo* CNVs of known pathogenic significance observed in a number of genomic disorders (Kirov et al., 2012) was a duplication at the WBS region at 7q11.23. Noteworthy, LIMK1 is among the 25 genes deleted in WBS (Tassabehji et al., 1999; Medrano-Fernández and Barco, 2016), however, the gene is duplicated in some patients with autism or schizophrenia.

The development of the instruments controlling transition between conformations might serve as a new therapeutic strategy for these human diseases. This requires the usage of simple models and the plausible one *is Drosophila* model for WBS, i.e., the mutant and spontaneous variants of the *agnostic* locus harboring the X-chromosome-linked LIMK1 gene (Savvateeva-Popova et al., 2004; Nikitina et al., 2014a). The region is involved in Kosikov duplication characterized by homology between the X-chromosome regions 11A and 12D and between 11B and 12E (Kosikov, 1936), the genomic material from 11A10-A11 and 11B3-11B9 mirrors each other (Savvateeva-Popova et al., 2002, 2004). It is a hot spot of chromosomal breaks, ectopic contacts, underreplication (Zhimulev et al., 1982; Belyaeva et al., 1998), and recombination (Hawley, 1980) facilitated by chemical mutagen ethyl methanesulfonate (EMS) (Xamena et al., 1985). According to current views (Medrano-Fernández and Barco, 2016), this region apparently belongs to topological domains or topologically associating domains (TADs).

Temperature-sensitive (ts) mutation *agn*^*ts*3^ showing ts-lethality at all stages of development at 29°C, but not in adulthood, was isolated in a screen for the X-chromosome EMS induced ts- mutations affecting cAMP metabolism (Savvateeva and Kamyshev, 1981; Medvedeva and Savvateeva, 1991). Adult *agn*^*ts*3^ flies demonstrate three diagnostic features of NDs (Hirsch, 2006): memory defects, locomotor impairments, and amyloid-like brain inclusions at normal temperature. HS treatment resulted in disappearance of these mutant manifestations (Medvedeva et al., 2008).

Besides, *agn*^*ts*3^ mutation leads to: (1) an increased level of LIMK1 and p-cofilin in the adult brain or 3rd instar larval salivary glands at 22°–25°C and a return to the wild type level at elevated temperature; (2) high rates of recombination modulated by temperature in the region of *agn*^*ts*3^ localization; (3) three-fold increase in frequency of ectopic contacts in the same region (Medvedeva et al., 2008). Thus, *agn*^*ts*3^ is a mutation not only changing the single-gene activity, but also the chromatin structure, and, as shown in this study, is expressed on the background of decreased miRNAs expression. Along these lines, recent findings indicate that 3D architecture of chromatin is involved in the transcriptional regulation of miRNAs (Chen et al., 2014). Also, a number of studies on different animal and plant species demonstrated that stress results in the modulation of miRNA levels. As shown in *Drosophila* (Funikov et al., 2016) strain-specific microRNA levels form a uniform microRNA pattern after HS. Besides, the different groups of such HS-sensitive miRNAs regulate functionally similar genes during the heat shock response (HSR).

In this study, we demonstrate that contrary to these recent findings, *agn*^*ts*3^ has a very unique miRNA expression signature and manifests different types of HSR: miRNAs expression might increase, decrease, or remain unchanged. This follows from the analysis of heat maps of micro RNAs expression and PCA (principal component analysis) in *agn*^*ts*3^ and the wild-type strains *Belin, Canton-S*, and *Oregon-R* (Figures 5, 6). Also, contrary to observed (Funikov et al., 2016), different strains may demonstrate an inverse HSR of same microRNAs. As for *agn*^*ts*3^, the overall level of microRNAs expression both under normal conditions and after HS is significantly lower than in other strains studied herein. However, the expression of certain microRNAs blocks increases after HS up to the wild type level (Table S3). Since HS restores learning acquisition and memory retention in *agn*^*ts*3^, the HS-induced increase in expression of specific microRNAs deserves a special attention: mir-1000 (negative regulation of glutamate secretion, neurotransmission), let-7, mir-8 (neuroblast development, regulation of NMJ development), members of the cluster mir-304 and mir-12 (regulation of smoothened signaling pathway). Similarly to our recent observation (Funikov et al., 2016), the expression levels of several clustered miRNAs respond to HS individually and independently of each other: in the cluster let-7, mir-100, mir-125 HS up-regulates let-7 expression, but down-regulates mir-100. Also, HS leads to down-expression of mir-277 cluster (mir-34, mir-317) and mir-306 cluster (mir-9c, mir-9b, mir-79). Noteworthy, these microRNAs belong to biomarkers of neurodegenerative diseases (NDs) (Maciotta et al., 2013) and all vertebrate miRNA families have representatives in *Drosophila* (Ibáñez-Ventoso et al., 2008). Since a single miRNA can regulate thousands of target genes, their deregulation is a major cause of NDDs, also termed as RNA disorders (Johnson et al., 2012). Moreover, among the 35–40 miRNAs highly abundant in the human CNS only six are the key players in chronic inflammatory NDDs. These are stress-regulated miRNA-7, miRNA-9, miRNA-34a, miRNA-125b, miRNA-146a, and miRNA-155 (Maciotta et al., 2013). The biomarker of PD is let-7/miR-184^*^ (Maciotta et al., 2013). Findings in *Drosophila* (Venderova et al., 2009), demonstrate that let-7 target gene is LRRK2 (leucine-rich repeat kinase2). It is involved in regulation of actin cytoskeleton and interacts with the protein products of partner genes of LIMK1, such as *Parkin* and *Pink*. In *Drosophila*, let-7 is sensitive to steroid hormones and determines the onset of neuron formation in the brain mushroom bodies (Chawla and Sokol, 2012; Kucherenko and Shcherbata, 2013). A biomarker for AD is miR-34. This steroid hormones-sensitive miRNA is considered to be a key regulator of age-related physiological changes, because its target genes include *tau, creb*, and *hsp70*. Therefore, miR34 is involved in negative regulation of aging and death of neurons (Ghosh et al., 2008; Maciotta et al., 2013; Feng et al., 2014). The remarkable role of miR-34 in development and disease (Rokavec et al., 2014) is explained by the existence of the p53/miR-34 axis. The tumor suppressor p53 responding to a myriad different types of stress (among them hypoxia) and contributing to the pathology of NDDs binds directly to response elements within the miR-34a and miR-34b/c promoters that contain inverted repeats creating local cruciform structures (Coufal et al., 2013).

As show in this study, additional binding site for mir-34 is created by the insertion of S-element from Tc1/mariner family in *agn*^*ts*3^ (Figure S3). The biomarker of HD is miR-36 (Maciotta et al., 2013). This individual manner of clustered miRNAs response to HS, specifically the miRNAs belonging to cluster 277–34, was also observed (Funikov et al., 2016). It could be, that similarly to recently revealed in *C. elegans* (Nehammer et al., 2015), this is a specific set of *Drosophila* miRNAs which play a crucial role during developmental survival and in behavioral functions after HS.

In this study we have found strain-specific INDEL polymorphisms in *LIMK1* sequence in the wild-type strains (*CS, OrR, Ber*) and in *agn*^*ts*3^ (Table 1, Table S1). The unique feature of *agn*^*ts*3^ compared to the wild-type strains is the presence of S-element from Tc1/mariner family located 456 bp downstream *LIMK1* transcription termination site (TTS). As shown in *Drosophila*, insertions of TEs downstream genes within 500 bp of TTS may efficiently suppress gene activity (Cridland et al., 2015). Notably, in humans members of Tc1/mariner superfamily posses numerous INDELs and serve as markers of a 1.7-kb recombination hot spot in genetic disorders such as Charcot–Marie–Tooth, Prader-Willi, Angelman, and Williams syndromes (Feschotte, 2008). Besides, the copies of this element located in multiple sites of *D.melanogaster* genome participate in the stimulation of homologous recombination between repeated sequences through the introduction of DBSs in DNA near to sites of strand exchange (Reiter et al., 1999). Probably, *agn*^*ts*3^ represents an example of a chimeric gene born by capture of the TE (Cordaux et al., 2006) during EMS-induced mutagenesis, since EMS serves as an amplifier of pre-existing natural variability (Ohnishi, 1977). Moreover, S-element in *agn*^*ts*3^ X:11AB region affects the chromatin structure changing the pattern of ectopic contacts and, thereby *LIMK1* expression level. Interestingly, as shown in this study, four miRNAs reside in the X-chromosome regions which form ectopic contacts with X:11AB region (Table S3). Two of them, dme-mir-304_5arm and dme-mir -12_5arm, both located in 13C7 are involved in positive regulation of smoothened signaling pathway, dme-mir-969_3arm (16F7) is involved in germ cell development and dme-mir-2535b_3arm (19F3) has yet unknown functions. Interestingly, smoothened, and type 6 serotonin receptor involved in stress response are found in cilia, actin-based subcellular structures present in a majority of cells including neurons. They are envisioned as the cellular “antennae” attuned for detecting a range of environmental signals including photons, odorants, morphogens, hormones, and mechanical forces (Qiu et al., 2016).

Another kind of explanation of *agn*^*ts*3^ ts-phenotypes and a support to a notion (Wells, 2007) that different environmental factors including HS, can cause transition of individual DNA segments from the linear B-DNA form to non-B DNA conformations comes from our previous findings (Lushnikov et al., 2014). First, unusual conformational DNA dynamics of 119 bp DNA fragment isolated from *agn*^*ts*3^ Intron 1 harboring 28 bp A/T-rich insertion has been found by physical methods of Brillouin light scattering upon heating the DNA sample till temperatures of DNA denaturation. Second, computer modeling of possible conformations which might be attained by the A/T rich 28 bp DNA insertion has shown that cruciforms, nodes and bubbles might evolve upon increasing temperature in 22°–37°C range. These structures occur *in vivo* during local strand separation required for replication, recombination regulation of gene expression, and nucleosome remodeling (Brázda et al., 2011; Kim and Kim, 2016).

In *agn*^*ts*3^ this insertion occurs together with the insertion of S-element and shows a partial similarity to its terminal repeats. This may cause pairing within *LIMK1*, changing its architecture (Figure 4) and thereby affecting *agn*^*ts*3^ *LIMK1* expression, at least in response to different temperature regimes (temperature sensitivity). As shown here (Figure 3), the inverted terminal repeats of *S-LIMK1* (Tc1/*mariner*) are nucleosome-free, as well as A/T rich 28 bp insertion in intron 1. A genome-wide analysis of conformational properties of naked DNA in yeast (Deniz et al., 2011) demonstrates that nucleosome positions adjacent to TSS (transcription start site) and TTS (transcription termination site) mostly depend on physical features of the naked DNA that govern equilibrium of its conformations. Therefore, it is not surprising that Palindrome analyzer (Brázda et al., 2016) reveals a huge prevalence in cruciform structures formed in *agn*^*ts*3^ *LIMK1* sequence homing both *S-LIMK1* (Tc1/*mariner*) and A/T rich 28 bp insertion in intron 1.

Moreover, as we have shown (Figure S1), that insertion of *S-LIMK1* (Tc1/mariner) creates additional 13 TF binding sites, such as Foxa2, Foxd3, Foxq1, and NFY, and promotes an appearance of additional br_Z1 binding sites (ecdysone-sensitivity) in *agn*^*ts*3^. Notably, spatial or temporal patterns of gene expression are set by the DNA cis-regulatory elements termed enhancers. They are enriched in TF binding sites that regulate gene activation and act from a distance to the TSS of their target genes. Besides, noncoding RNAs can be transcribed from enhancers (Plank and Dean, 2014). TF binding itself can realize in nucleosome-depleted stretches of DNA through interaction with other TFs in the same or other chromosome, thus providing a topological basis for transcription regulation (Li et al., 2012).

Therefore, spatial localization of S-element and 28 bp A/T-rich insertion sharing partial homology with terminal repeats of the transposon is capable of providing such a topological basis in *agn*^*ts*3^ which may be considered as “a conformational mutant.”

Taken together, our results help to highlight the DNA conformational dynamics as a point of application of therapeutic strategies for neurological diseases and genomic disorders caused by INDELs, Transposable Elements of the Tc1/*mariner* Superfamily and microRNAs.

## Author contributions

Conceived and designed the experiments: AM, EN, JD, OZ, ME, and ES. Performed the experiments: AZ, GZ, AK, AM, EN, ET, JD, DK, OZ, SF, and SR. Analyzed the data: AZ, GZ, AK, AM, EN, ET, JD, DK, OZ, SF, SR, ME, and ES. Contributed reagents/materials/analysis tools: GZ, VB, ME, and ES. Wrote the paper: AZ, AM, JD, GZ, SF, ME, and ES.

### Conflict of interest statement

The authors declare that the research was conducted in the absence of any commercial or financial relationships that could be construed as a potential conflict of interest.

## Supplementary material

Figure S1 Transcription factor binding sites in *LIMK1* and *S-LIMK1* sequences (Mapper data). Red square: TF binding site. Two red rectangles: TF dimer binding site. Green square: the variable TF binding site and nucleotide polymorphism.

Figure S2 miRNA processing genes expression for *agn*^*ts*3^ and *Ber*. Quantitative real-time PCR data. Relative expression levels of miRNA biogenesis pathway genes in studied *Drosophila* strains; mean values ± error bars for three replicates are represented. *agn*^*ts*3^ genes with statistically significant differences (*P* ≤ 0.05) in expression level compared to *Ber* are shown by asterisk.

Figure S3 miRNA binding sites within polymorphic areas of *LIMK1*. miRNA binding sites are shown by triangles, only for extensive polymorphic areas. Ex—exon, i—intron.

Table S1 Strain-specific LIMK1 polymorphisms. Ins—insertion, del—deletion. The base number and nucleotides are shown for genomic sequence. For strain-specific sequnces, only the variants different from genomic sequence are shown. Red color: the unique agnts3 strain-specific polymorphism.

Table S2 The detected strain-specific polymorphisms in LIMK1 sequence and their possible effects.
