# Supplementary figures and images for: Drosophila Model for the Analysis of Genesis of LIM-kinase 1-Dependent Williams-Beuren Syndrome Cognitive Phenotypes: INDELs, Transposable Elements of the Tc1/Mariner Superfamily and MicroRNAs
Source: Front Genet. 2017 Sep 20;8:123. doi: 10.3389/fgene.2017.00123 (PMC5611441; doi:10.3389/fgene.2017.00123)

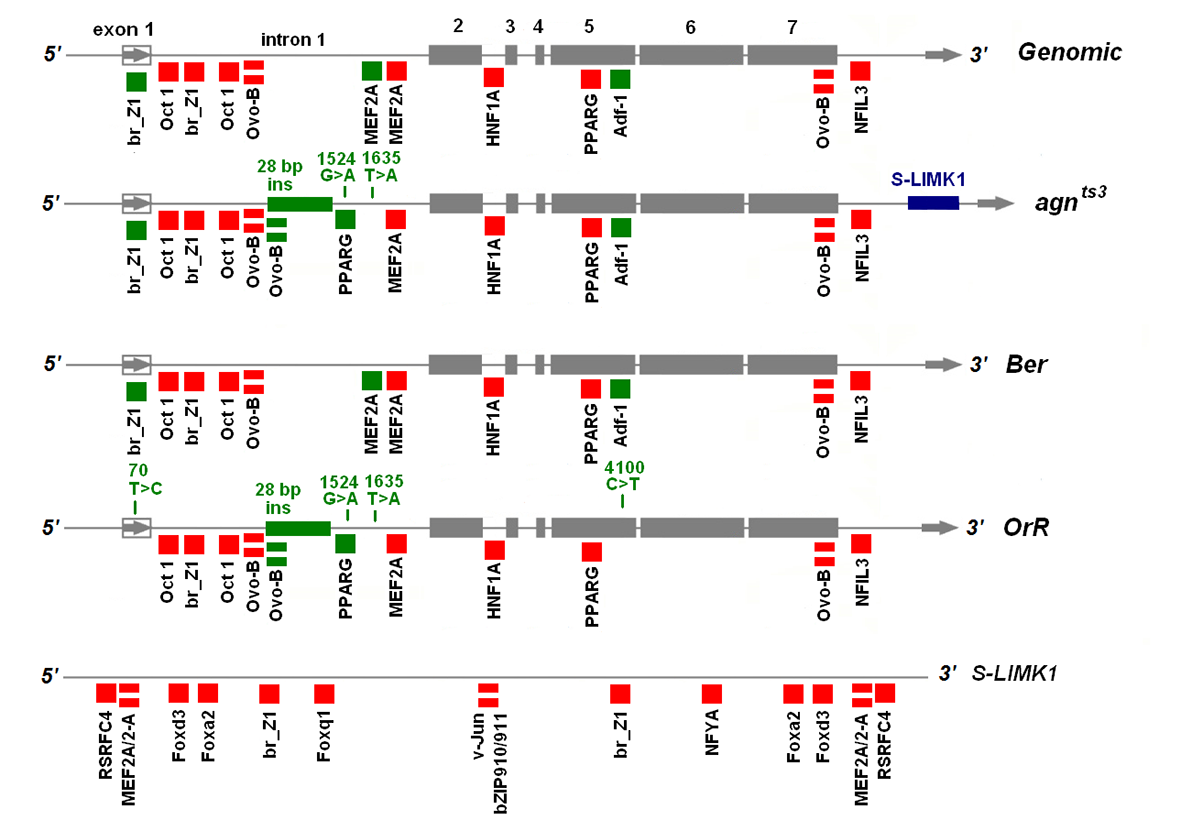

Supplement: Figure S1 — Transcription factor binding sites in LIMK1 and S-LIMK1 sequences (Mapper data). Red square: TF binding site. Two red rectangles: TF dimer binding site. Green square: the variable TF binding site and nucleotide polymorphism. [file Image1.TIF]

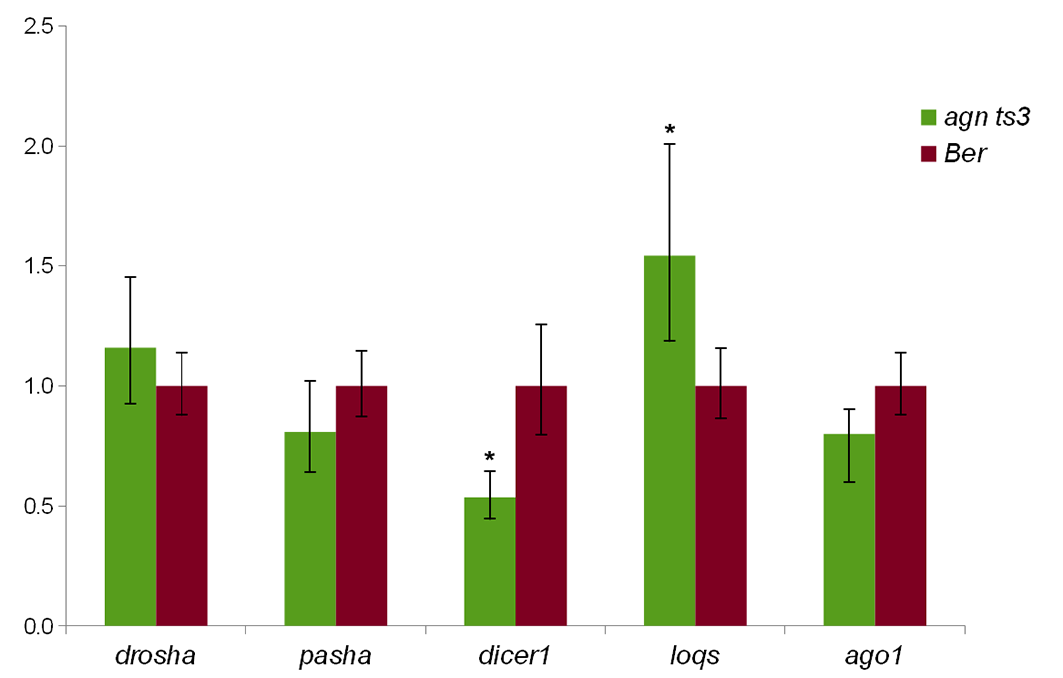

Supplement: Figure S2 — miRNA processing genes expression for agnts3 and Ber. Quantitative real-time PCR data. Relative expression levels of miRNA biogenesis pathway genes in studied Drosophila strains; mean values ± error bars for three replicates are represented. agnts3 genes with statistically significant differences (P ≤ 0.05) in expression level compared to Ber are shown by asterisk. [file Image2.TIF]

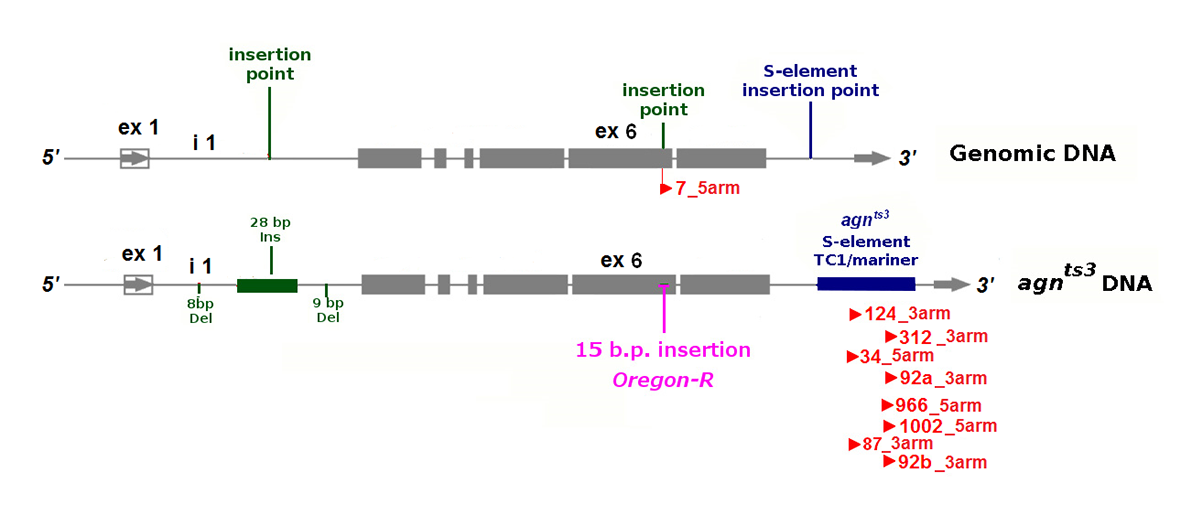

Supplement: Figure S3 — miRNA binding sites within polymorphic areas of LIMK1. miRNA binding sites are shown by triangles, only for extensive polymorphic areas. Ex—exon, i—intron. [file Image3.TIF]
