# Supplementary material for: Drosophila Model for the Analysis of Genesis of LIM-kinase 1-Dependent Williams-Beuren Syndrome Cognitive Phenotypes: INDELs, Transposable Elements of the Tc1/Mariner Superfamily and MicroRNAs
Source: Front Genet. 2017 Sep 20;8:123. doi: 10.3389/fgene.2017.00123 (PMC5611441; doi:10.3389/fgene.2017.00123)
Supplement: Table S1 — Strain-specific LIMK1 polymorphisms. Ins—insertion, del—deletion. The base number and nucleotides are shown for genomic sequence. For strain-specific sequnces, only the variants different from genomic sequence are shown. Red color: the unique agnts3 strain-specific polymorphism. [file Table1.PDF]

Base pair	*Genomic*	*Canton-S*	*agnostic ts3*	*Oregon-R*	*Berlin*	*Gene regions*
-200	C		T		C/T	
-176	A	A/G		G		
-71	A	A/T	T	T	A/T	
-67	G	G/T	T	T	G/T	
-60	A	A/T		T		
9	C	C/T	T	T	C/T	0 – 262: exon 1
70	T			C/T		
72	T			C/T		
109	C	C/T	T	T	C/T	
335-336		ins A			ins A	263 – 1708: intron 1
414-421		-/del 8 bp	del 8bp	del 8bp		
472	C	C/T		T		
493	C	C/A	A	A		
506	T	T/C	C	C		
564	C		A	A		
565	G	G/A	del G	del G		
578	A	A/G	A/G	G		
634	A	A/T	T	T	A/T	
636	C	C/A	C/A	A	C/A	
657	C	C/T		T		
675	A	A/G	A/G	G	A/G	
766	G	G/A	G/A	A	G/A	
785	T		T/G			
842	A		A/T		A/T	
853	G	G/A	G/A	A	G/A	
873	G	G/A		A		
1180	G		A	G	G/A	
1262	C	C/T	T	T	C/T	
1346-1347		/ins A	ins A	ins A		
1352-1353		/ins 28 bp	ins 28bp	ins 28bp		
1378	A	A/G	G	G		
1386-1388	CAA	CAA/AGC	AGC	AGC		
1403-1411		-/del 9bp	del 9bp	del 9bp		
1449	A	A/G	G	G		
1469	A		G	A/G		
1524	G	G/A	A	A		
1635	T	T/A	A	A		
1891	G	G/A	A	A	G/A	1709 – 2352: exon 2
2104	C		T		C/T	
2293	C	T	T	C/T		
2386	T	T/C	C	T/C		2353 – 2871: intron 2
2393	A	A/T	T	A/T		2509 – 2669: exon 3
2463-2464		ins 2T	ins 2T	ins 2T		2670 – 3025: intron 3
2496	T	T/C	T/C		T/C	2845 – 2937: exon 4
2504	T	C	T/C	C		
2667	G				G/A	
2804	G					
2822	G	G/A				
2869	T	T	C	C	C	

2953	G			G/A	A	2938 – 3025: intron 4
2962	T			T/C	T/C	
2967	G			G/A	G/A	
2994	T			T/G	T/G	
3001-3002	TA	TA/CT	CT	CT	TA/CT	
3069	T	T/C	C	T/C		3026-4223: exon 5
3281	G	G/A		G/A		
3317	G	G/A		A		
3350	C	C/G		G		
3413	T	T/C		C		
3476	T	T/C		C		
3506	C	C/A		A		
3671	A	G		G		
3731	A	A/G		G		
3828	G	G/A		A		
3941	C	C/G		G		
4031	C	C/T		T		
4046	C	C/G		G		
4100	C	C/T		T		
4226	A	A/G		G		4224 – 4292: intron 5
4237	A	A/T		T		
4241	T	T/C		C		
4346	C	C/T		T		4293 – 5883: exon 6
4385	T	T/C		C		
4391	T	T/C		C		
4421	G	G/A		A		
4631	G	G/A		A		
4784	A	A/G		G		
4826	C		G		C/G	
5020	C	C/T		T		
5045	A	A/G		G		
5063	A	A/C		C		
5111	A	G		G		
5297	G	A		A		
5396	G	T		T		
5402	A	G		G		
5522				ins 15 bp		
5633	C	C/T	T	T	C/T	
5900	C			C/G		5884 – 5943: intron 6
6035	C	C/T				5944 – 7808: exon 7
6131	T	T/G				
6136	G	G/A				
6153	A	A/C				
6167	C	C/T				
6257	A	A/G				
8264			ins *S-LIMK1*			
