# Supplementary material for: Drosophila Model for the Analysis of Genesis of LIM-kinase 1-Dependent Williams-Beuren Syndrome Cognitive Phenotypes: INDELs, Transposable Elements of the Tc1/Mariner Superfamily and MicroRNAs
Source: Front Genet. 2017 Sep 20;8:123. doi: 10.3389/fgene.2017.00123 (PMC5611441; doi:10.3389/fgene.2017.00123)
Supplement: Table S2 — The detected strain-specific polymorphisms in LIMK1 sequence and their possible effects. [file Table2.PDF]

Positi-on (bp)	Region (region boundaries)	Polymorphisms					Possible effects (strain)
		**Geno-mic**	***CS***	***agn**^{ts3}*	***OrR***	***Ber***	
-71	71 bp upstream exon 1; Hb binding site	A	A/T	T	T	A/T	*mRNA A/C/E* transcription decrease (*agn*^{ts3}, OrR)
9	Exon 1 (0 – 225/262); M1 binding site	C	C/T	T	T	C/T	*mRNA A/C/E* transcription decrease (*agn*^{ts3}, *OrR*)
414 – 421	Intron 1 (226/263 – 1708)		–/8 bp del	8 bp del	8 bp del		Unknown
785	Intron 1	T	T	T/G	T	T	Unknown
1346	Intron 1, TATA box		–/A	A	A	–	Transcription regulation
1352 – 1353	Intron 1	–	–/28 bp ins	28 bp ins	28 bp ins	–	Nucleosome formation probability decrease (*agn*^{ts3}, *OrR*)
1403 – 1411	Intron 1		-/9 bp del	9 bp del	9 bp del		Unknown
2504	Exon 2 (2510 – 2669); HSF binding site	T	T	C	C	T	*mRNA D* transcription decrease (*agn*^{ts3}, *OrR*)
3828	Exon 5 (3026 – 4223)	G	G/A	G	A	G	Val(493)Ile polymorphism (*CS*, *OrR*)
5020	Exon 6 (4293 – 5883)	C	C/T	C	T	C	Pro(867)Leu polymorphism (*CS*, *OrR*)
5063	Exon 6	A	A/G	A	G	A	Glu(881)Asp polymorphism (*CS*, *OrR*)
5522 – 5523	Exon 6	–	–	–	15 bp ins	–	5 AA insertion (1034-1035) into LIMK1 C-domain, dme-miR-7-5p site disruption (*OrR*)
6136	Exon 7	G	G/A	G	G	G	Ser(1219)Asn polymorphism (*CS*)
6153	Exon 7 (5944 – 6767)	A	A/C	A	A	A	Thr(1225)Pro polymorphism (*CS*)
8264	456 bp downstream exon 7	–	–	S-LIMK1	–	–	Chromosomal architecture changes (*agn*^{ts3})
